# Primary Squamous Cell Carcinomas Arising in Intracranial Epidermoid Cysts: A Series of Nine Cases and Systematic Review

**DOI:** 10.3389/fonc.2021.750899

**Published:** 2021-10-26

**Authors:** Pengcheng Zuo, Tao Sun, Yi Wang, Yibo Geng, Peng Zhang, Zhen Wu, Junting Zhang, Liwei Zhang

**Affiliations:** ^1^ Department of Neurosurgery, Beijing Tiantan Hospital, Capital Medical University, Beijing, China; ^2^ Department of Neurosurgery, Beijing Chao-Yang Hospital, Capital Medical University, Beijing, China; ^3^ China National Clinical Research Center for Neurological Diseases, Beijing, China; ^4^ Beijing Key Laboratory of Brain Tumor, Beijing, China

**Keywords:** intracranial epidermoid cysts, malignant transformation, gross total resection, radiotherapy, the cerebellopontine angle

## Abstract

**Objective:**

Primary squamous cell carcinomas (PSCCs) arising in intracranial epidermoid cysts (IECs) are very rare, and their management and prognostic factors remain unclear. This study aimed to enunciate the clinical features and suggest a treatment protocol based on cases from the literature and the cases from our institution.

**Methods:**

The clinicoradiological data were obtained from nine patients with PSCCs arising in IECs, who underwent surgical treatment at Beijing Tiantan Hospital between July 2012 and June 2018. We also searched the PubMed database using the keywords “epidermoid cyst(s)” or “epidermoid tumor(s)” combined with “malignant” or “malignancy” or “intracranial” or “brain” or “squamous cell carcinoma” between 1960 and 2020. Risk factors for overall survival (OS) were evaluated in the pooled cohort.

**Results:**

The mean age of our cohort was 51.2 ± 8.3 years (range: 39–61 years), which included eight males and one female. Gross total resection (GTR) was achieved in three patients, while non-GTR was achieved in six patients. Radiotherapy was administered to five patients. After a median follow-up of 16.7 ± 21.6 months (range: 3–72 months), eight patients died with a mean OS time of 9.75 ± 6.6 months (range: 3–23 months). In the literature between 1965 and 2020, 45 cases of PSCCs arising in IECs were identified in 23 males and 22 females with a mean age of 55.2 ± 12.4 years. GTR, non-GTR, and biopsy were achieved in six (13.3%), 36 (80%), and three (6.7%) cases, respectively. After a mean follow-up of 12.7 ± 13.4 months (range: 0.33–60 months), 54.1% (20/37) patients died, and recurrence occurred in 53.6% (15/28) patients. A multivariate analysis demonstrated that postoperative radiotherapy (p = 0.002) was the only factor that favored OS. The Kaplan–Meier analysis showed that, compared with no radiotherapy (median survival time: 4 months), radiotherapy (median survival time: 24 months) had significantly prolonged OS (p = 0.0011), and GTR could not improve OS (p = 0.5826), compared with non-GTR. The 1-year OS of patients with or without radiotherapy was 72.5% or 18.2%, respectively.

**Conclusion:**

Malignant transformation of IEC into PSCC was prevalent in elderly patients, with slight male predominance. GTR of previous benign IECs is recommended. For remnant benign IECs, close follow-up should be performed. Postoperative radiotherapy for PSCCs could bring survival benefit. GTR of these malignant intracranial tumors is difficult when they involve important brain structures. Future studies with larger cohorts are necessary to verify our findings.

## Introduction

Intracranial epidermoid cysts (IECs) are benign tumors that typically develop in the cerebellopontine angle (CPA) region (1). The IEC represents about 0.2%–1.8% of all brain tumors, and its malignant transformation into SCC is a rare occurrence (2, 3). Most of these tumors are regarded as congenital lesions arising from heterotopic epithelial cells misplaced in the neural tube when the latter separates from the ectoderm between the third and fifth weeks of embryonal life (4–6). Ernst reported the first case of such malignant transformation in 1912 (2), and a few cases that met the Garcia criteria have been subsequently reported. Garcia et al. (7) defined the criteria for the malignant transformation of IECs as follows: the tumor had to be restricted to the intracranial or intradural compartment without invasion or extension beyond the dura or cranial bones. Also, there must be no extension or invasion through the intracranial orifices; no communication or connection with the middle ear, air sinuses, or sella turcica; and no evidence of nasopharyngeal tumor. According to Hamlat (8), primary squamous cell carcinoma (PSCC) was classified into five groups: 1) initial malignant transformation of a benign cyst, 2) malignant transformation from a remnant cyst, 3) malignant transformation of a dermoid and epithelial cyst, 4) malignant transformation with leptomeningeal carcinomatosis, and 5) other malignancies arising from benign cysts. Herein, we described nine cases of PSCCs arising in IECs at our center and also reviewed relevant literature from the PubMed database.

## Methods

Nine patients who underwent surgical treatment were pathologically confirmed to have PSCCs at Beijing Tiantan Hospital between July 2012 and June 2018. According to Hamlat (8), PSCC could arise in an untreated benign IEC or a remnant benign IEC. Among our nine cases, four had a history of previous resection of benign ECs. The other five patients did not have a history of surgery and were considered as having malignant transformation of IECs due to coexistence of benign ECs and squamous cell carcinomas in the lesion without the evidence of metastatic diseases. The following information was collected: age, sex, symptoms, symptom duration, imaging characteristics, extent of tumor excision, pathological results, treatment, and prognosis. Pre- and postoperative MRI scans were performed to determine the extent of tumor resection, which was defined as gross total resection (GTR) and non-GTR. The follow-up was performed by telephone interview every six months. This study was approved by the Beijing Tiantan Hospital Research Ethics Committee.

Then we searched the PubMed database using the keywords “epidermoid cyst(s)” or “epidermoid tumor(s)” combined with “malignant” or “malignancy” or “intracranial” or “brain” or “squamous cell carcinoma” between 1960 and 2020. A total of 45 articles in English language that met the Garcia criteria were included.

The risk factors for overall survival (OS) were evaluated with univariate and multivariate Cox regression analyses of a total of 46 cases (including 37 cases from the literature and nine cases from our center). The survival curves were performed by the Kaplan–Meier method. Analyses were performed using SPSS Statistical Package software (version 22.0, IBM Corp.), with the significance set at p < 0.05.

## Results

### Cases From Our Institution

The cases from our institute included eight males and one female with a mean age 51.2 ± 8.3 years (range: 39–61 years). The duration of symptoms ranged from 2 to 12 months with a mean length of 9.33 ± 3.74 months. The preoperative symptoms included headache (n = 3), facial numbness (n = 3), trouble coughing (n = 3), diplopia (n = 2), limb weakness (n = 2), facial paralysis (n = 2), tinnitus (n = 1), ataxia (n = 1), ptosis (n = 1), hearing loss (n = 1), and vomiting (n = 1). Five patients experienced natural malignant transformation of benign intracranial ECs, and four patients suffered malignant transformation of remnant benign IECs. The interval from EC was 3–28 years (**Table 1**). MRI showed that the tumors were most commonly located in the CPA region (n = 8), and only one case was located in the suprasellar region. On contrast-enhanced MRI, enhancement was observed in all nine cases, and peritumoral edema was observed in six (66.7%) cases. All patients accepted surgical treatment. GTR was achieved in three patients, and non-GTR was achieved in six patients. Radiotherapy was administered to five patients, of whom one patient accepted gamma knife therapy, one patient accepted proton beam therapy, and three patients accepted external beam radiation. After a mean follow-up of 16.7 ± 21.6 months, eight patients died, and the mean OS was of 9.75 ± 6.6 months. Among them, seven patients died of tumor recurrence, and one patient died of intracranial infection. Postoperative pathological examination revealed squamous cell carcinoma. Immunohistochemical staining showed that the Ki67 proliferative indexes ranged from 10% to 90% (**Table 1**).

**Table 1:** General characteristics of nine cases in our institute.

Case	Age (years)	Sex	Tumor location	Symptoms	Symptom duration (months)	Enhancement	Peritumoral edema	Surgical history	Treatment	Ki-67	Recurrence	Dead and alive	Follow-up time (months)	Interval from EC (years)	Extent of resection for previous ECs
1	39	M	CPA	Headache, diplopia, facial numbness	12	Yes	Yes	No	Non-GTR	–	Yes	Dead	4	–	–
2	54	F	Suprasellar region	Headache, diplopia, vomiting	2	Yes	Yes	No	Non-GTR	40%	Yes	Dead	4	–	–
3	43	M	CPA	Facial numbness	6	Yes	Yes	No	Non-GTR+PB	30%	Yes	Alive	72	–	–
4	44	M	CPA	Trouble coughing, limb weakness	6	Yes	Yes	Yes	GTR+LRT	60%	Yes	Dead	10	3	Non-GTR
5	51	M	CPA	Facial numbness, facial paralysis, tinnitus	12	Yes	Yes	No	Non-GTR+LRT	30%	Yes	Dead	3	–	–
6	48	M	CPA	Trouble coughing, facial paralysis, ataxia	10	Yes	No	Yes	GTR	10%	No	Dead	9	15	Non-GTR
7	61	M	CPA	Ptosis, trouble coughing	12	Yes	No	Yes	Non-GTR+LRT	50%	Yes	Dead	23	28	Non-GTR
8	61	M	CPA	Hearing loss, limb weakness	12	Yes	Yes	No	GTR	90%	Yes	Dead	12	–	–
9	60	M	CPA	Headache	12	Yes	No	Yes	Non-GTR+GKS	60%	Yes	Dead	13	5	Non-GTR

F, female; M, male; CPA, cerebellopontine angle; GTR, gross total resection; Non-GTR, non-gross total resection; LRT, local radiotherapy; GKS, gamma knife surgery; PB, proton beam.

### Cases From the Literature

A total of 45 patients (23 were males and 22 were females) diagnosed with intracranial PSCCs were identified between June 1960 and July 2020 (**Table 2**). The mean age of patients was 55.2 ± 12.4 years (range 30–83 years). On contrast-enhanced MRI or CT, enhancement was observed in all 39 available cases, and the tumors were typically located in the CPA region (n = 23). Peritumoral edema was observed in 18 (42.9%) cases. Surgical resection was performed as GTR in six patients and non-GTR in 36 patients. Twenty-three patients had a surgical history, and the pathological results were benign ECs. The interval from EC was 0.17–40 years with a mean time of 9.4 ± 11.8 years. Postoperative radiotherapy was administered to 21 patients (46.8%), of whom three patients accepted gamma knife therapy. Chemotherapy was administered to five patients (8.5%). After a mean follow-up of 13.0 ± 13.3 months (range: 0.33–60 months), 52.6% (20/38) patients died, while 53.6% (15/28) patients suffered from recurrence (**Table 3**).

**Table 2:** Literature review of PSCCs arising in IECs studies from 1960 to 2020.

Authors and year	Sex/age (years)	Location	Surgical history	Treatment (Gy)	Recurrence	Enhancement	Peritumoral edema	Dead or alive	Follow-up time (months)	Interval from EC (years)	Extent of resection for previous ECs
Davidson et al., 1960 (9)	M/46	Frontal	No	Non-GTR+LRT	No	NA	NA	Alive	NA	–	–
Fox et al., 1965 (10)	M/43	Midline of skull base	Yes	Surgery	NA	–	NA	Dead	1.5	7.75	Non-GTR
Dubois et al., 1981 (11)	M/53	Fourth ventricle	No	Non-GTR+LRT (50)	NA	Yes	NA	Dead	2	–	–
Lewis et al., 1983 (12)	F/54	Suprasellar region	No	Surgery	Yes	Yes	Yes	Dead	1	–	–
Giangaspero et al., 1984 (13)	M/45	Paraventricular	No	Surgery+LRT (45)	Yes	Yes	Yes	Dead	8	–	–
Goldman et al., 1987 (14)	F/59	Pineal region	Yes	Non-GTR+LRT (50)	Yes	Yes	Yes	Alive	36	33	Surgery
Nishiura et al., 1989 (15)	M/38	CPA	Yes	Non-GTR+CT	No	Yes	Yes	Alive	24	0.58	Non-GTR
Knorr et al., 1991 (16)	M/74	CPA	Yes	Non-GTR	Yes	Yes	Yes	Dead	1.75	1.08	Surgery
Tognetti et al., 1991 (17)	F/67	Frontal and temporal areas	Yes	Non-GTR	NA	Yes	No	Dead	1	31	Non-GTR
Acciarri et al., 1993 (5)	M/62	Parasellar region	No	Non-GTR	NA	Yes	No	Dead	0.33		–
Nishio et al., 1995 (18)	M/57	CPA	No	Non-GTR+LRT (50)	No	Yes	No	Alive	30	–	–
Uchino et al., 1995 (19)	M/57	CPA	No	Non-GTR+LRT (60)	No	Yes	No	Alive	4	–	–
Bayindir et al., 1996 (20)	F/67	Lateral ventricle	Yes	Non-GTR	No	NA	No	Alive	36	0.83	Non-GTR
Murase et al., 1999 (21)	F/50	CPA	Yes	Non-GTR+RS (14)+CT	No	Yes	No	Alive	60	10	GTR
Asahi et al., 2001 (22)	F/55	CPA	Yes	Non-GTR	Yes	Yes	No	Dead	3	13	NA
Link et al., 2002 (23)	F/57	CPA	Yes	Non-GTR+LRT (45)+SR (15)	Yes	Yes	Yes	Dead	32	1	Non-GTR
Hamlat et al., 2003 (3)	F/54	Lateral ventricle and temporal	Yes	CT	NA	Yes	No	Dead	16	0.25	Non-GTR
Shirabe et al., 2003 (24)	M/49	Brainstem	No	Biopsy+LRT (60)	NA	Yes	Yes	Dead	24	–	–
Michael et al., 2005 (25)	M/45	Prepontine area	No	Non-GTR	Yes	Yes	No	Dead	12	–	–
Tamura et al., 2006 (26)	F/56	CPA	Yes	Non-GTR+GSK (Total 27)	Yes	Yes	No	Alive	11	8	Non-GTR
Agarwal et al., 2007 (27)	M/45	Posterior fossa	No	Surgery	NA	NA	No	NA	–	–	–
Kodama et al., 2007 (28)	M/67	CPA	No	Non-GTR	Yes	Yes	Yes	Dead	12	–	–
Pagni et al., 2007 (1)	F/65	Pineal region	No	Surgery	NA	Yes	No	NA	NA	–	–
Kim et al., 2008 (4)	F/72	Brainstem	No	Non-GTR+IMRT (54)	No	Yes	No	Alive	12	–	–
Ge et al., 2009 (29)	M/44	Temporal	Yes	Surgery	NA	NA	No	NA	–	6	GTR
Hao et al., 2010 (6)	F/61	Temporal	No	Non-GTR	Yes	Yes	Yes	Dead	1.2	6	–
Kano et al., 2010 (30)	F/64	CPA	Yes	Non-GTR+LRT (50)	Yes	Yes	No	Dead	22	15	Non-GTR
Nakao et al., 2010 (31)	F/74	CPA	Yes	Non-GTR+LRT (46)	No	Yes	No	Alive	17	20	Surgery
Shah et al., 2010 (32)	M/36	CPA	No	Surgery+RT	No	Yes	Yes	Alive	8	–	–
Lakhdar et al., 2011 (33)	M/52	Brainstem and cerebellum	Yes	GTR+LRT (50)	No	Yes	Yes	Alive	1	0.5	GTR
Chon et al., 2012 (34)	M/43	CPA	Yes	Non-GTR+GSK (25)	Yes	Yes	Yes	Alive	13	0.42	Non-GTR
Feng et al., 2014 (35)	M/42	CPA	No	Non-GTR	No	Yes	No	Alive	6	–	–
Vellutini et al., 2014 (36)	F/42	CPA	Yes	Non-GTR	NA	Yes	No	Dead	1.33	1.5	Non-GTR
Chourmouzi et al., 2015 (37)	F/39	CPA	Yes	GTR	NA	Yes	Yes	NA	NA	NA	–
Ding et al., 2016 (38)	F/55	Temporal	No	Surgery	Yes	Yes	No	Dead	6	–	–
Pikis et al., 2016 (39)	M/77	CPA	Yes	Non-GTR+LRT (55)	NA	Yes	No	Dead	6	0.75	Non-GTR
Raheja et al., 2016 (40)	F/37	CPA	No	Biopsy+CSR+CT	Yes	Yes	No	Dead	11	–	–
Ozutemiz et al., 2017 (41)	M/64	Lateral ventricle	No	Surgery	Yes	Yes	Yes	Alive	3	23	–
Roh et al., 2017 (2)	F/53	CPA	No	GTR+IMRT (67.2)	No	Yes	No	Alive	20	–	–
Seif et al., 2017 (42)	M/83	Posterior fossa	Yes	GTR	NA	NA	No	NA	NA	0.17	GTR
Solanki et al., 2017 (43)	F/47	CPA	Yes	Non-GTR	NA	Yes	Yes	Dead	1.5	1	Non-GTR
Cuoco et al., 2019 (44)	M/71	CPA	Yes	Non-GTR+SR (25)	NA	Yes	Yes	Alive	NA	40	Surgery
Demuth et al., 2019 (45)	F/67	CPA	No	Surgery+CT	NA	Yes	Yes	Alive	NA	–	–
Fereydonyan et al., 2019 (46)	M/30	CPA	Yes	GTR+LRT	No	Yes	Yes	Alive	24	5	Non-GTR
Gerges et al., 2019 (47)	F/65	Pineal region	No	GTR	No	Yes	No	Alive	1.5	–	–

NA, Not available; IMRT, intensity-modulated radiotherapy; CSR, craniospinal irradiation; SR, stereotactic radiosurgery; RS, radiosurgery; PSCCs, primary squamous cell carcinomas; IECs, intracranial epidermoid cysts; EC, epidermoid cyst; GTR, gross total resection; LRT, local radiotherapy; CPA, cerebellopontine angle; Non-GTR, non-gross total resection.

**Table 3:** Summary of clinical characteristics of PSCCs arising in IECs from literature and our institute.

Variable	Prior studies (n = 45)		Our series	Overall
	No. of available cases	Value		
Interval from EC, years	24	9.4 ± 11.8	12.8 ± 11.4	9.9 ± 11.6
Mean age, years	45	55.2 ± 12.4	51.2 ± 8.3	54.5 ± 11.8
Sex (M/F)	45	23/22	8/1	31/23
CPA region	45	23 (51.1%)	8 (88.9%)	31 (57.4%)
Enhancement	39	39 (100%)	9 (100%)	48 (100%)
Peritumoral edema	42	18 (42.9%)	6 (66.7%)	24 (47.1%)
GTR	45	6 (13.3%)	3 (33.3%)	9 (16.7%)
Radiotherapy	45	21 (46.7%)	5 (55.6%)	26 (48.1%)
Chemotherapy	45	5 (11.1%)	0 (0)	5 (9.3%)
Recurrence	29	15 (51.7%)	8 (88.9%)	23 (60.5%)
Death	40	20 (50%)	8 (88.9%)	28 (57.1%)
Mean FU, months	37	12.7 ± 13.4	16.7 ± 21.6	13.5 ± 15.1

Values are presented as the number of patients (%), and mean values are presented as the mean ± SD.

FU, follow up; PSCCs, primary squamous cell carcinomas; IECs, intracranial epidermoid cysts; EC, epidermoid cyst; CPA, cerebellopontine angle; GTR, gross total resection.

### Statistical Analysis of Prognostic Factors for Overall Survival

**Figure 1:**
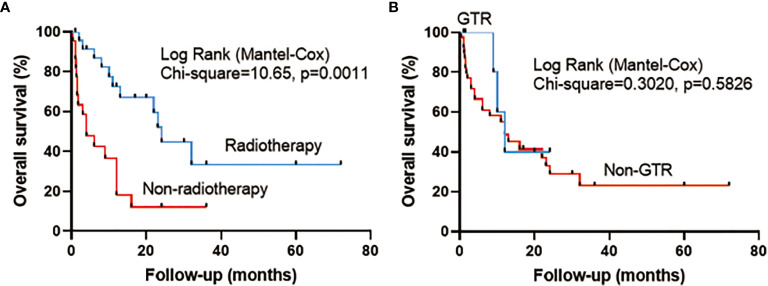
Survival curve analysis (log-rank test) illustrating the different overall survival (OS) rates in pooled cases between **(A)** radiotherapy and no radiotherapy and between **(B)** gross total resection (GTR) and non-gross total resection (Non-GTR).

The mean follow-up in the pooled cohort (46 cases) was 13.5 ± 15.1 months. The results of univariate Cox regression analysis (including sex, age, surgical history, tumor location, peritumoral edema, extent of tumor resection, radiotherapy, and chemotherapy) showed that compared with no postoperative radiotherapy, postoperative radiotherapy had significantly prolonged OS (HR 0.298, 95% CI 0.138–0.648, p = 0.002). Other factors, including sex (HR 1.086, 95% CI 0.509–2.318, p = 0.831), age (HR 1.139, 95% CI 0.541–2.398, p = 0.732), surgical history (HR 0.728, 95% CI 0.342–1.548, p = 0.409), tumor location (HR 0.582, 95% CI 0.274–1.233, p = 0.158), GTR (HR 0.718, 95% CI 0.216–2.389, p = 0.589), peritumoral edema (HR 1.115, 95% CI 0.514–2.419, p = 0.783), and chemotherapy (HR 0.463, 95% CI 0.109–1.961, p = 0.296) were not significant. Then we added sex, age, GTR, and radiotherapy to the multivariate Cox regression analysis. Radiotherapy (HR 0.300, 95% CI 0.138–0.652, p = 0.002) was the only protective factor (**Table 4**). The Kaplan–Meier analysis showed that compared with no radiotherapy (median survival time: 4 months), radiotherapy (median survival time: 24 months) significantly prolonged OS of patients (p = 0.0011) (**Figure 1A**) , and GTR could not improve OS (p = 0.5826) (**Figure 1B**). The 1-year OS in patients with or without radiotherapy was 72.5% or 18.2%, respectively.

**Table 4:** Cox regression model for risk factors predicting OS.

Variables	Number of patients	Death (%)	Univariate analysis		Multivariate analysis	
			HR (95% CI)	p value	HR (95% CI)	p-Value
Sex						
Male	26	12 (60)	Reference		Reference	
Female	20	16 (61.5)	1.086 (0.509–2.318)	0.831	1.086 (0.487–2.423)	0.840
Age, years						
<55	24	15 (62.5)	Reference		Reference	
≥55	22	13 (59.1)	1.139 (0.541–2.398)	0.732	1.091 (0.501–2.376)	0.826
Surgical history						
Yes	23	14 (60.9)	Reference			
No	23	14 (60.9)	0.728 (0.342–1.548)	0.409		
Tumor location						
CPA	28	16 (57.1)	Reference			
Others	18	12 (66.7)	0.582 (0.274–1.233)	0.158		
GTR						
Yes	7	3 (42.9)	Reference		Reference	
No	39	25 (64.1)	0.718 (0.216–2.389)	0.589	0.691 (0.203–2.346)	0.553
Peritumoral edema						
Yes	21	13 (61.9)	Reference			
No	23	13 (56.5)	1.115 (0.514–2.419)	0.783		
Radiotherapy						
Yes	24	11 (45.8)	Reference		Reference	
No	22	17 (77.3)	0.298 (0.138–0.648)	0.002*	0.300 (0.138–0.652)	0.002*
Chemotherapy						
Yes	4	2 (50)	Reference			
No	42	26 (61.9)	0.463 (0.109–1.961)	0.296		

OS, overall survival; CPA, cerebellopontine angle; GTR, gross total resection.

*p < 0.05 was considered statistically significant.

## Illustrative Case

### Case 8

**Figure 2:**
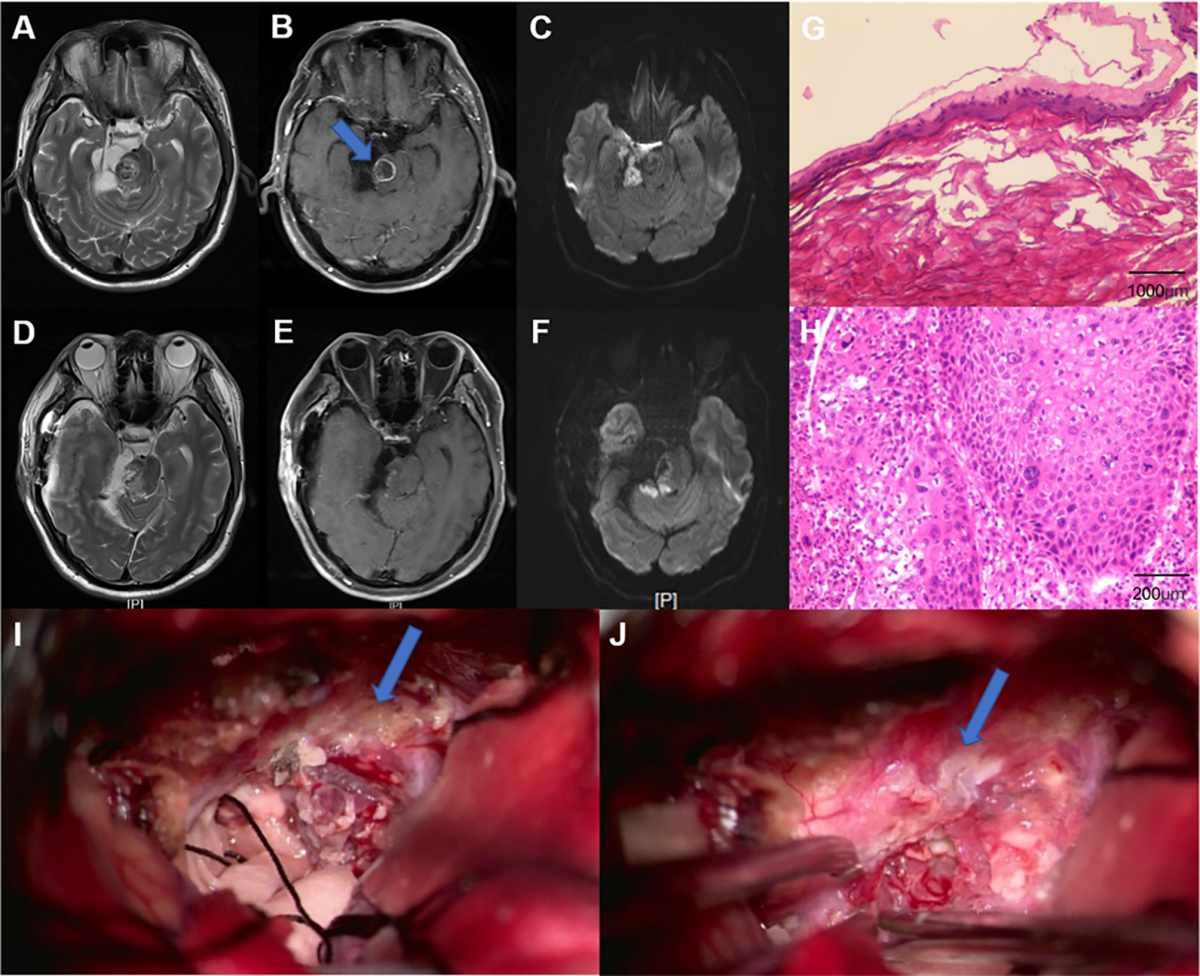
**(A–C)** Preoperative MRI. **(A)** Axial, T2-weighted MRI showed a heterogeneous signal of the lesion located at the right cerebellopontine angle (CPA) region. **(B)** Axial, contrast-enhanced MRI revealed relatively regular, ring-like enhanced lesion in the right brainstem. **(C)** Axial, diffusion-weighted imaging (DWI) showed a heterogeneous signal of the lesion. **(D–F)** Postoperative MRI. The solid mass was totally resected. Pathological results indicated a benign EC **(G)** (specimen from outside the brainstem) (H&E, ×40) and squamous cell carcinoma **(H)** (specimen from inside the brainstem) (H&E, ×200). **(I, J)** Images during the surgery. **(I)** The resection of the benign epidermoid cyst (as pointed by an arrow). **(J)** A part of the capsule that adhered tightly to the brainstem (as pointed by an arrow).

A 61-year-old male presented with dizziness and hearing loss for over 1 year and developed left hemiparesis for 2 weeks. Neurological examination revealed dysfunction of right cranial nerve VIII and weakness of left limb. A T2-weighted MRI showed a heterogeneous signal of the lesion located at the right CPA region. Diffusion-weighted imaging (DWI) revealed a high-signal-intensity lesion located in the right CPA cistern, prepontine cistern, and right cisterna ambiens and low signal intensity in the right pons. A T1-weighted gadolinium-enhanced MRI showed an intra-axial ring-like enhanced mass in the right brainstem, while the high-signal-intensity lesion on DWI showed no enhancement. Peritumoral edema was also observed (**Figures 2A–C**). A right sub-temporal craniotomy was performed. During surgery, the tumor (outside the brainstem) was found to be visually white and pearly-like with less blood supply. The tumor outside the brainstem was easily removed, and the tumor inside the brainstem was also totally resected (**Figures 2I, J**). His postoperative course was uneventful. Postoperative MRI showed no tumor residue (**Figures 2D–F**). The result of pathological examination showed EC (outside the brainstem) and SCC (inside the brainstem) (**Figures 2G, H**). Immunohistochemistry revealed that the Ki67 proliferation index was approximately 90%, which may indicate aggressive biological behavior of the tumor. The patient also underwent a whole-body positron emission tomography, and the results did not show any evidence of metastatic disease. A diagnosis of PSCC arising in IEC was made. We advised the patient to consult radiotherapists for further treatment. The patient refused to undergo radiotherapy and died of tumor recurrence 12 months after surgery.

## Discussion

The IECs, sometimes termed as intracranial pearly tumors or cholesteatomas, are rare lesions that represent approximately 7% of the CPA tumors, and the malignancy of IECs is an extreme occurrence (2, 28). At our institute, 1,095 cases of IECs were pathologically confirmed between July 2012 and June 2018. Among them, nine cases showed PSCCs arising in IECs, and the malignancy of IECs was approximately 0.82% at our center. In this study, we discussed the clinical features of intracranial PSCCs and proposed a treatment protocol based on 54 cases (including 45 cases from the literature and nine cases from our institution).

It was reported that these tumors often affected elderly patients with female predominance (33); however, all nine cases from our hospital and the pooled 45 cases showed a slight male predominance (57.4%). Similar to previous studies (26, 33), the interval to malignant transformation was relatively long, ranging from 0.17 to 40 years with a mean time 9.9 ± 11.6 years. Malignant transformation usually occurred within the primary location of the lesions, which were primarily located in the CPA area but also found in the parapontine, intraventricular, and parasellar regions (26, 43). The growth rate of IECs was linear rather than exponential, and rapid progression of symptoms and signs may indicate the malignant transformation of the lesion (31). PSCC could arise in a natural malignant transformation of IEC or a remnant benign IEC. Of the pooled analysis, 27 cases experienced natural malignant transformation of IEC, and another 27 cases had a surgical history, and there was no statistical difference in OS between the two groups (p = 0.409). Although the first case of malignancy of IEC was reported over a century ago, the mechanisms underlying this transformation remain unclear. Some potential mechanisms include a chronic inflammatory response due to repeated cystic rupture or subtotal resection of the cystic wall (4). In this study, 21 patients (84%) with non-GTR and four (16%) patients with GTR suffered malignant transformation of previous benign IECs. Hence, we hypothesized that non-GTR of previous benign IECs might be a risk factor for the malignant transformation. The mechanism of malignancy may be the recurrent inflammatory stimulation of the residual tumor. Close follow-up should be performed for those who did not achieve GTR of benign IECs. Repeated cystic rupture may be another explanation for the malignant transformation of ECs. In 2010, Hao et al. reported a natural malignant transformation of an IEC without a history of surgery and suggested that spontaneous rupture of the EC may contribute to the malignant transformation (6). We found that some benign ECs had thickened capsules. Postoperative pathological examination showed epithelial dysplasia in these capsules. We speculated that the epithelial dysplasia may lead to PSCC.

Radiologically, typical benign IECs appear as low-density lesions in the subarachnoid space without contrast enhancement on CT or MRI and hyperintense on DW-MRI. The malignant transformation of intracranial epithelial cysts appears as apparent enhancement by contrast medium on CT or T1-weighted MRI and hypointense on DWI (30, 31). In this study, significant enhancement on MRI or CT was observed in all cases (48 cases were available). Although some enhanced lesions can exist adjacent to or within benign epidermoid cysts without malignant transformation (28), appearance of a significant enhancement in epidermoid cyst should alert the neurosurgeon of a malignant transformation. Peritumoral edema was also found in these tumors (47.1%). Interestingly, univariate analysis showed that absence of peritumoral edema (p = 0.783) could not predict a better OS. Histopathologically, the epidermoid cyst wall comprises benign squamous epithelium, and the cysts have keratin debris and squamous epithelium but lack malignant cells. The primary intracranial squamous cell carcinomas have poorly differentiated epithelial cells with pleomorphic nuclei, and they display stromal invasion. Among eight patients from our institute, the Ki67 index was relatively high, ranging from 10% to 90%, which indicated a relatively aggressive disease course. Our study failed to confirm the positive efficacy of GTR for survival benefit. Limited cases with GTR (n = 7) may cause potential bias of pooled analysis. In addition, GTR of intracranial PSCCs was controversial. Given the adherent nature of the cyst wall, it may adhere tightly to some important structures, such as brainstem or the adjacent cranial nerves, which would make GTR impossible. The modalities of adequate chemotherapy have been examined in various studies, but the effectiveness remains uncertain (8). Radiotherapy after the surgery of intracranial PSCCs seems effective (31). Nagasawa et al. reviewed 36 cases of epidermoid tumors with malignant transformation and compared survival outcomes between the surgery alone group and the surgery plus radiotherapy group. OS of patients treated with postoperative radiotherapy was 6.1 months longer than that of patients treated with surgery alone (48). The survival rate was increased in those receiving radiotherapy than in those who achieved no further treatment after the surgery (4). The stereotactic radiosurgery has also been used in some cases with satisfactory survival benefits (23). Our study also confirmed the positive effect of postoperative radiotherapy with larger cohorts (p = 0.002), and the mean OS of patients with radiotherapy was 20 months longer than that of patients with no radiotherapy. It is noted that some patients did not accept radiotherapy because of severe neurological dysfunction after surgery. We recommend that when the tumor involves the brainstem and/or cranial nerves, the operation should not be aggressive. The proton beam therapy might be effective. Chen et al. reported a 43-year-old male who accepted proton beam therapy after surgery with no evidence of tumor recurrence for 2 years (49). Coincidentally, the patient accepted surgery at our hospital in September 2015, and we continued to follow up this patient. Through telephone interview, he was uneventful until February 2021, but 1 month later, he presented with dizziness, headache, and dysfunction of cranial nerves IX, X and XI; and the MRI scan revealed tumor recurrence. He has been alive for 72 months now. To the best of our knowledge, this case has the longest OS in the literature.

In addition, it was reported that IECs could transform into melanoma. Kaif et al. (50) reported a 26-year-old female with malignant melanoma arising in a cerebellopontine EC; and Meng et al. (51) described a case of fulminant leptomeningeal carcinomatosis from a malignant melanoma arising in a cerebellopontine epidermoid cyst. Contrast enhancement on MRI and rapid progression of clinical symptoms may indicate malignant transformation of IECs.

## Conclusion

Malignant transformation of IEC into PSCC is very rare and has a poor clinical outcome. We described nine cases of malignant IECs at our institute over a period of 6 years and reviewed the relevant literature. These lesions often affect elderly patients. Contrast enhancement on MRI and rapid progression of clinical symptoms may indicate malignant transformation of IECs. Non-GTR of previously benign IECs may have potential risk for malignant transformation. For remnant benign IECs, close follow-up should be performed. Postoperative radiotherapy for PSCCs could bring survival benefit. GTR of these malignant intracranial tumors is difficult when they involve critical brain structures. Future studies with larger cohorts are necessary to verify these findings.

## Data Availability Statement

The original contributions presented in the study are included in the article/supplementary material. Further inquiries can be directed to the corresponding author.

## Author Contributions

PCZ: writing—original draft and conceptualization. TS: formal analysis and investigation. YW: literature review. YG: follow-up. PZ: literature review. ZW: methodology and resources. JZ: methodology and resources. LZ: writing—review and editing, and supervision. All authors contributed to the article and approved the submitted version.

## Funding

This study was funded by Multicenter clinical big data study and multi-path tumorigenesis mechanisms and precision treatment research on brainstem glioma (JINGYIYAN2018-7) and Special Fund of the Pediatric Medical Coordinated Development Center of Beijing Hospitals Authority (XTYB201822).

## Conflict of Interest

The authors declare that the research was conducted in the absence of any commercial or financial relationships that could be construed as a potential conflict of interest.

## Publisher’s Note

All claims expressed in this article are solely those of the authors and do not necessarily represent those of their affiliated organizations, or those of the publisher, the editors and the reviewers. Any product that may be evaluated in this article, or claim that may be made by its manufacturer, is not guaranteed or endorsed by the publisher.
